# Measuring agreement between decision support reminders: the cloud vs. the local expert

**DOI:** 10.1186/1472-6947-14-31

**Published:** 2014-04-10

**Authors:** Brian Edward Dixon, Linas Simonaitis, Susan M Perkins, Adam Wright, Blackford Middleton

**Affiliations:** 1 Department of BioHealth Informatics, Indiana University School of Informatics and Computing, Indiana University-Purdue University Indianapolis, 410 W. St., Suite 2000, Indianapolis, IN 46202, USA; 2 Center for Biomedical Informatics, Regenstrief Institute, Inc, Indianapolis, IN USA; 3 Center for Health Information and Communication, Department of Veterans Affairs, Veterans Health Administration, Health Services Research and Development Service CIN 13–416, Indianapolis, IN USA; 4 Department of Internal Medicine, Indiana University School of Medicine, Indianapolis, IN USA; 5 Department of Biostatistics, Indiana University, School of Medicine, Indianapolis, IN USA; 6 Indiana University Cancer Center, Indianapolis, IN USA; 7 Indiana Clinical Translational Science Institute, Indianapolis, IN USA; 8 Harvard Medical School, Boston, MA USA; 9 Division of General Medicine, Brigham and Women’s Hospital, Boston, MA USA; 10 Partners HealthCare, Boston, MA USA; 11 Department of Biomedical Informatics, Vanderbilt University Medical Center, Nashville, TN USA

**Keywords:** Decision support systems, Clinical decision making, Computer-assisted knowledge management, Statistical data analysis, Preventive health services

## Abstract

**Background:**

A cloud-based clinical decision support system (CDSS) was implemented to remotely provide evidence-based guideline reminders in support of preventative health. Following implementation, we measured the agreement between preventive care reminders generated by an existing, local CDSS and the new, cloud-based CDSS operating on the same patient visit data.

**Methods:**

Electronic health record data for the same set of patients seen in primary care were sent to both the cloud-based web service and local CDSS. The clinical reminders returned by both services were captured for analysis. Cohen’s Kappa coefficient was calculated to compare the two sets of reminders. Kappa statistics were further adjusted for prevalence and bias due to the potential effects of bias in the CDS logic and prevalence in the relative small sample of patients.

**Results:**

The cloud-based CDSS generated 965 clinical reminders for 405 patient visits over 3 months. The local CDSS returned 889 reminders for the same patient visit data. When adjusted for prevalence and bias, observed agreement varied by reminder from 0.33 (95% CI 0.24 – 0.42) to 0.99 (95% CI 0.97 – 1.00) and demonstrated almost perfect agreement for 7 of the 11 reminders.

**Conclusions:**

Preventive care reminders delivered by two disparate CDS systems show substantial agreement. Subtle differences in rule logic and terminology mapping appear to account for much of the discordance. Cloud-based CDSS therefore show promise, opening the door for future development and implementation in support of health care providers with limited resources for knowledge management of complex logic and rules.

## Background

Attention to preventive care can protect patients from developing serious health conditions and supports the triple aim of reducing health care costs while improving the quality and efficiency of care delivery [1]. Numerous public and private organizations, including most professional medical societies, publish guidelines that describe recommendations for proper preventive care. Unfortunately, patients receive recommended preventive care just 54.9% of the time [2]. Too often busy clinicians treat acute medical problems, lacking the time required to address a patient’s preventive care.

Evidence demonstrates that computerized provider order entry (CPOE) with clinical decision support (CDS) can improve the delivery of preventive care [3-8]. Given evidence of the potential value CDS holds for achievement of the triple aim, U.S. health care policymakers advocate wider adoption and use of CPOE with CDS [9-11]. Stage 2 Meaningful Use criteria from the Centers for Medicare and Medicaid Services, the federal agency tasked with incentivizing the adoption of electronic health record (EHR) systems, place greater emphasis on CDS, escalating the number of required decision support rules linked to specific quality indicators [12].

Policies like Meaningful Use are likely necessary as many hospitals and clinics failed to adopt CDS prior to their passage. Currently just 15% of the 5795 U.S. hospitals have a “basic” electronic health record system, and only 4.4% of hospitals report implementing “core” functionalities of the meaningful use criteria which include CDS [13]. Furthermore, adoption of CDS is typically found in larger, urban academic medical centers which can mandate use by providers [14]. Although 86% of all U.S. hospitals are community hospitals, just 6.9% of community hospitals have reported having a basic clinical information system [15]. Rates are equally poor for other types of hospitals, with just 6% of long-term acute care hospitals, 4% of rehabilitation hospitals, and 2% of psychiatric hospitals reporting the use of a basic electronic health record system [16].

Implementation of CDS to comply with federal regulations, however, is not sufficient to ensure its use. Several studies highlight that certain forms of CDS are turned off or ignored following implementation [17-19]. A fundamental barrier for many providers is the creation and curation of preventive care rules, alerts, and reminders; a process referred to as knowledge management (KM) [20-22]. KM is challenging as it requires significant investment in human and infrastructure resources to ensure that the knowledge base supporting CDS is accurate and up-to-date [23-25].

Local experts within an institution are often charged with KM tasks such as designing CDS-based preventive service reminders. Often these experts are asked to translate preventive service guidelines from national information sources to the local CDS system. While these local experts are familiar with the terminologies and policies at their institution and therefore often successful, their efforts are laborious and require continuous review, updates, and management. A recent survey found that, while KM tasks necessary to “customize” CDS are routinely performed in both large as well as small-to-medium sized community hospitals, the level of effort required to customize CDS prior to implementation was greater than expected [26]. The task of KM is therefore daunting, and it remains unclear how to scale the financial, technical, and human capital necessary to support CDS across all U.S. hospitals. Therefore new methods and models for KM and dissemination of knowledge for CDS are needed to support national efforts towards achieving meaningful use and the triple aim.

Given the need for scalable KM across an increasing landscape of hospitals with CDS, we sought to compare preventive reminders created using traditional, local expert KM processes with reminders developed collaboratively for a cloud-based CDS system operating across a consortium of independently managed hospitals. In 2008, the Regenstrief Institute joined the Clinical Decision Support Consortium (CDSC) [27], which seeks “to assess, define, demonstrate, and evaluate best practices for knowledge management and clinical decision support in healthcare information technology at scale – across multiple ambulatory care settings and EHR technology platforms” [28]. The CDSC, funded by the U.S. Agency for Healthcare Research and Quality (AHRQ), is based at Partners Healthcare, but involves a growing array of CDS stakeholders.

To compare local expertise driven CDS methods with those of the CDSC, we executed parallel sets of preventive service guidelines: one set implemented locally by Regenstrief experts and, independently, another set implemented in the cloud-based CDSC web service by knowledge engineers at another institution. Although the two implementations were different, the preventive guidelines which they covered were the same. The study is unique because it directly compares the outcome of preventive service guidelines enacted at separate institutions for the same set of patient data. It is further unique in that it examines a novel modality of CDS where KM and execution of rules are performed “in the cloud” to reduce burden on hospitals in their efforts to implement and adopt CDS.

## Methods

This research was conducted principally at Eskenazi Health (formerly Wishard Health Services), a large, urban safety net provider in Marion County, Indiana. Eskenazi Health includes a 315-bed hospital and 11 community health centers. Almost 1.4 million outpatient visits annually take place at these facilities. Eskenazi Health is closely integrated with the Indiana University School of Medicine and includes a large presence from medical students, resident physicians, and other health professionals in training.

Regenstrief Institute, Inc. is a research institution closely affiliated with Eskenazi Health, and provides Eskenazi clinicians with order entry and decision support services. Since the 1970s, Regenstrief has provided KM for the various alerts, reminders, and displays that support patient care at Eskenazi Health. Non-urgent preventive care reminders (e.g., recommendations for mammograms or cholesterol testing) are written in the CARE language and delivered to the physician at the beginning of each patient visit [29].

In July 2011, we began a 6-month feasibility study to incorporate CDSC preventive care reminders into the CareWeb information system used in Eskenazi Health community health centers. Patient enrollment was limited to those patients who arrived for a scheduled outpatient visit for three part-time physicians practicing at two health centers. We limited the current investigation to the final three months (October 1 to December 31, 2011) of this feasibility study during which the receipt, integration, and logging of ECRS preventive care reminders were fully operational. The study obtained ethics approval and a waiver of written informed consent from the Indiana University Institutional Review Board (Study No. 1111007478).

Every time a patient arrived at a clinic for a visit with one of the physicians, an electronic arrival message was generated by the front desk registration system. This arrival message triggered the automated assembly of a standards-based continuity of care document (CCD) through a query of the patient’s electronic health records. A limited data set was encoded into the CCD as dates of service were required for successful execution of CDS logic. However, other patient identifiers including name, medical record number, and date of birth were de-identified. The CCD was sent to the CDSC cloud-based service at Partners [30,31]. The term ‘cloud-based’ refers to a specific set of characteristics and services available on-demand across a network from a pool of computing resources [32]. Prior articles from the CDSC describe its cloud-based architecture and implementation [33,34].

After processing by the CDSC service, preventive care reminders (if applicable) were included in the response message returned from Partners to Regenstrief where they were written to a table in Regenstrief’s enterprise CDS infrastructure. When a physician viewed the patient’s record in the CareWeb information system, these preventive care reminders were displayed. As previously mentioned, this feasibility study was limited to the eleven preventive care reminders used in the pilot project shown in Table 1.

**Table 1:** List of the 11 preventive care reminders provided by CDSC web service

**ID**	**Conditions triggering rule**	**Message displayed**
1	[Diabetes] AND [no HgbA1c result within last 6 months]	Diabetic patient is overdue for HgbA1c measurement (recommended every 6 months)
		• Order HgbA1c now.
2	[Diabetes] AND [last HgbA1C result between 5 and 6 months ago]	Diabetic patient is almost due for HgbA1c measurement (recommended every 6 months)
		• Order HgbA1c now.
3	[Diabetes] AND [last HgbA1c result between 3 and 5 months ago] AND [greater than 8%]	Last HgbA1c was greater than 8% and over 3 months ago (recommended every 3 months in poorly controlled patient)
		• Order HgbA1c now.
4	[Diabetes] AND [no established renal disease] AND [no microalbumin result in the last 11 months]	Diabetic patient is due for urine microalbumin/creatinine ratio measurement (recommended yearly)
		• Order malb/creat ratio now.
5	[Diabetes] AND [chronic renal disease] AND [not ESRD] AND [not taking an ACE-I] AND [not taking an ARB] AND [no contraindication to ACE-I]	Diabetic patient with renal disease, consider starting angiotensin-converting enzyme inhibitor (ACE-I).
		• Start ACE-I.
6	[Diabetes] AND [chronic renal disease] AND [not ESRD] AND [not taking an ACE-I] AND [not taking an ARB] AND [contraindication exists to ACE-I] AND [no contraindication to ARB]	Diabetic patient with renal disease, contraindications to ACE-I present, consider starting angiotensin-2 receptor antagonist (ARB).
		• Start ARB.
7	[Diabetes] AND [last eye exam over 11 months ago]	Diabetic patient is due for ophthalmologic exam (recommended yearly)
		• Document the eye exam.
		• Refer to Ophthalmologist.
		• Refer to Optometrist.
8	[Diabetes] AND [last foot exam over 11 months ago]	Diabetic patient is due for foot exam (recommended yearly)
		• Document the foot exam.
		• Refer to Podiatrist.
9	No blood pressure within last 12 months	Patient is overdue for blood pressure assessment (recommended yearly)
		• Document the blood pressure.
10	[CAD] AND [not on any antiplatelet medication] AND [contraindication exists to antiplatelet therapy]	Patient has CAD or equivalent, consider starting anti-platelet therapy, but potential contraindications exist.
		• Start aspirin.
		• Start clopidogrel.
11	[CAD] AND [not on any antiplatelet medication] AND [no contraindication to antiplatelet therapy]	Patient has CAD or equivalent, recommend starting anti-platelet therapy.
		• Start aspirin.
		• Start clopidogrel.

HgbA1c = Hemoglobin A1C. ESRD = End stage renal disease. ACE-I = Angiotensin-converting enzyme inhibitor. ARB = Angiotensin receptor blocker. CAD = Coronary artery disease.

At the conclusion of the study period, we tabulated which preventive care reminders were delivered by the CDSC web service for each patient visit (defined as the combination of the patient’s medical record number and the visit date).

Then we gathered eleven corresponding CARE rules developed at Regenstrief. The eleven rules in each set (the CDSC set and the Regenstrief CARE set) attempt to achieve the same result: encode the logic for the preventive care reminders in Table 1. However, the underlying details differ greatly. CDSC rules are written in the language specified for the IBM/ILOG rules engine; Regenstrief CARE rules are written for a custom-built rules engine based on the VMS operating system. Furthermore, CDSC rules rely on concepts coded in standard vocabularies (SNOMED CT, RxNORM, NDFRT, and LOINC) whereas CARE rules expect all concepts to be coded using Regenstrief’s local term dictionary.

The corresponding CARE rules were executed retrospectively for each of the patient visits in this study, relying on the data available for that patient on the date of that visit. We tabulated which preventive care reminders were generated by the CARE rule engine for each patient visit.

For each of the eleven reminders, we created a 2 × 2 frequency table and compared the cloud-based CDSC rules with the locally-crafted CARE rules for agreement with respect to the delivery (‘Yes’) or absence (‘No’) of a preventive care reminder. Four outcomes were possible: both rules delivered a reminder; only the CDSC rule delivered a reminder; only the CARE rule delivered a reminder; or neither rule delivered a reminder. Observed agreement (P_0_) is the proportion of times both the CDSC rule and the CARE rule agreed on ‘Yes’ or ‘No’.

The standard measure of agreement in a 2 × 2 frequency table is Cohen’s Kappa coefficient (κ). Kappa adjusts the observed agreement by the agreement expected by chance. However, if no further adjustments are made, Kappa can be deceptive, because it is sensitive to both the bias in reporting ‘Yes’ between the two rules, if any exists, and the prevalence of ‘Yes’ relative to ‘No’ in the sample.

The Bias Index (BI) measures the difference in the proportion of ‘Yes’ between the CDSC rules and the CARE rules. The Prevalence Index (PI) measures the difference in proportions between ‘Yes’ and ‘No’ overall (using only cases where both rules agreed). We adjusted the Kappa both for bias and for prevalence by calculating the Prevalence-Adjusted Bias-Adjusted Kappa (PABAK), in accordance with the methodology described by Byrt, Bishop and Carlin [35]. PABAK values were interpreted according to the guidelines for Kappa provided by Landis and Koch: 0.81 – 1.00: almost perfect agreement; 0.61 – 0.80: substantial agreement; 0.41 – 0.60: moderate agreement; 0.21 – 0.40: fair agreement; and 0.01 – 0.20: slight agreement [36]. In addition, we generated 95% confidence intervals for each value of PABAK using a bootstrap algorithm with 10,000 bootstrap samples [37].

We also compared the demographic data for the patients in the study sample to the total year 2011 clinic volume. A two-sample t-test for age, and chi-square tests for ethnicity, gender, and insurance status were performed. A p-value of < 0.05 was considered significant. SAS version 9.3 (Cary, NC) was used for all analyses.

## Results

### Patient demographics

During the three-month analysis period, 405 patient visits occurred. A total of 372 distinct patients were seen during visits to the three providers. Table 2 illustrates demographic data for the patients in the study sample, as well as the total year 2011 clinic volume. The study sample did not differ from the total clinic volume on ethnicity or gender, but was older on average, more likely to have Medicare insurance, and less likely to have Wishard Advantage insurance (a managed care program providing medical care to residents of Indianapolis with incomes less than 200% of the federal poverty level).

**Table 2:** Demographics of study patients compared with overall clinic population

**Characteristic**	**Study patients**		**Clinics**	
	**N = 372**		**N = 15572**	
	**Mean (SD)**		**Mean (SD)**	
Age	52.6 (13.9)		49.6 (14.9)	
	N	(%)	N	(%)
Ethnicity				
African-American	209	(56.2%)	8902	(57.2%)
White	117	(31.5%)	4427	(28.4%)
Latino	3	(0.8%)	224	(1.4%)
Other	12	(3.2%)	457	(2.9%)
Unknown	31	(8.3%)	1562	(10.0%)
Gender				
Female	217	(58.3%)	9680	(62.2%)
Male	155	(41.7%)	5892	(37.8%)
Primary insurance (first visit)				
Wishard advantage	153	(41.1%)	7441	(47.8%)
Medicare	119	(32.0%)	3596	(23.1%)
Medicaid	47	(12.6%)	2322	(14.9%)
Self-pay	30	(8.1%)	878	(5.6%)
Commercial	23	(6.2%)	1331	(8.5%)
Other	0	(0%)	4	(<1%)

### Observed agreement

During the three-month period, a total of 965 preventive care reminders were delivered by the cloud-based CDSC rules engine. For those same patient visits, 889 reminders were generated by locally-crafted CARE rules. These raw counts are compared in Table 3. Observed agreement (P_0_) varies from 0.66 to 0.99.

**Table 3:** Raw counts of preventive care reminders delivered by the cloud-based CDSC rules and the locally-crafted CARE rules

**Reminder**	**Both rules**	**CDSC rule, not CARE**	**CARE rule, not CDSC**	**Neither rule**	**P**_**0**_
	**N**	**N**	**N**	**N**	
	**(%)**	**(%)**	**(%)**	**(%)**	
1. Overdue for A1c	85 (21%)	20 (5%)	7 (72%)	293	0.93
2. Almost due for A1c	11 (3%)	1 (<1%)	2 (97%)	391	0.99
3. Recent A1c was over 8	4 (1%)	4 (1%)	15 (4%)	382 (94%)	0.95
4. Due for microalbumin screening	110 (27%)	15 (4%)	32 (8%)	248 (61%)	0.88
5. Renal disease, consider ACE inhibitor	12 (3%)	34 (8%)	44 (11%)	315 (78%)	0.81
6. Renal disease, contraindications for ACE inhibitor, consider ARB	1 (<1%)	0 (0%)	3 (1%)	401 (99%)	0.99
7. Due for eye exam	204 (50%)	12 (3%)	8 (2%)	181 (45%)	0.95
8. Due for foot exam	140 (35%)	27 (7%)	7 (2%)	231 (57%)	0.92
9. Due for blood pressure	84 (21%)	127 (31%)	9 (2%)	185 (46%)	0.66
10. CAD, consider anti-platelet	35 (9%)	8 (2%)	72 (18%)	290 (72%)	0.80
11. CAD, consider anti-platelet, but contraindications exist	2 (<1%)	29 (7%)	2 (<1%)	372 (92%)	0.92

P0 = observed agreement, the proportion of times both CDSC rule and CARE rule agreed. A1C = Hemoglobin A1C. ACE Inhibitor = Angiotensin converting enzyme inhibitor. ARB = Angiotensin receptor blocker. CAD = Coronary artery disease.

### Prevalence-adjusted bias-adjusted kappa

Kappa statistic is calculated and shown for the preventive care reminders in Table 4. Also shown are the Bias Index (BI), Prevalence Index (PI), and the Prevalence-adjusted Bias-adjusted Kappa (PABAK). The unadjusted Kappa statistic varies from 0.10 to 0.90, suggesting little agreement in Rule 11 (K = 0.10), Rule 5 (K = 0.13), and Rule 3 (K = 0.28). When adjusted for prevalence and bias, PABAK varies from 0.33 (95% CI 0.24 – 0.42) to 0.99 (95% CI 0.97 – 1.00).

**Table 4:** Unadjusted kappa statistic, as well as prevalence-adjusted bias-adjusted kappa

**Reminder**	**K**	**BI**	**PI**	**PABAK**	**95% CI PABAK**
1. Overdue for A1c	0.82	-0.03	-0.51	0.87	0.82 – 0.91
2. Almost due for A1c	0.88	0.00	-0.94	0.99	0.97 – 1.00
3. Recent A1c was over 8	0.28	0.03	-0.93	0.91	0.86 – 0.95
4. Due for microalbumin screening	0.74	0.04	-0.34	0.77	0.70 – 0.83
5. Renal Disease, consider ACE inhibitor	0.13	0.02	-0.75	0.61	0.54 – 0.69
6. Renal disease, contraindications for ACE inhibitor, consider ARB	0.40	0.01	-0.99	0.99	0.97 – 1.00
7. Due for eye exam	0.90	-0.01	0.06	0.90	0.86 – 0.94
8. Due for foot exam	0.82	-0.05	-0.22	0.83	0.78 – 0.89
9. Due for blood pressure	0.34	-0.29	-0.25	0.33	0.24 – 0.42
10. CAD, consider anti-platelet	0.37	0.16	-0.63	0.60	0.53 – 0.68
11. CAD, consider anti-platelet, but contraindications exist	0.10	-0.07	-0.91	0.85	0.79 – 0.90

K = Kappa statistic. BI = Bias Index. PI = Prevalence Index. PABAK = Prevalence-adjusted Bias-adjusted Kappa. CI = Confidence Interval. A1C = Hemoglobin A1C. ACE Inhibitor = Angiotensin converting enzyme inhibitor. ARB = Angiotensin receptor blocker. CAD = Coronary artery disease.

Using the Landis and Koch interpretation, the adjusted Kappa statistic (PABAK) demonstrates almost perfect agreement for 7 of the 11 preventive care reminders. Two more reminders (reminders 4 and 5) can be interpreted as substantially in agreement. The remaining two reminders (reminders 9 and 10) demonstrate fair or moderate agreement.

## Discussion

Using a limited set of preventive care reminders, we compared the results of CDS logic execution from a remote CDS web service with the results returned from a locally developed and maintained CDS infrastructure. Using the Kappa statistic, with adjustments for prevalence and for bias, we found a high level of agreement between the two sets of results. Strong agreement is auspicious for future development of cloud-based CDS that can support centralized knowledge management functions associated with operational CDS systems.

Our institution, like many other urban as well as community hospitals, has previously relied on decision support rules implemented and maintained locally. In the case of Eskenazi Health, these were carefully developed and maintained by local clinical informatics experts. Other institutions may purchase such rules directly from a vendor and install them in their local information system [38]. With either approach, institutions are challenged by constrained resources and substantial expenses if they seek to continue maintaining and expanding their own decision support infrastructure [24,26,38-40].

Cloud-based CDS represents a completely new model for delivering advice and guidelines to the point of care. In the current study, patient data at Eskenazi Health in Indiana were packaged into a standard envelope (the CCD document) using standard vocabulary identifiers. These data were sent to a distant, cloud-based web service hosted in Massachusetts. The decision support engine in the cloud generated reminders based on local patient data, and delivered the reminders to the local EHR system, where they were integrated for use by local clinicians.

This remote web service was not custom-built just for this transaction. The CDS infrastructure supporting the CDSC extended the CDSS which previously provided similar services to clinicians using the Longitudinal Medical Record at Partners HealthCare System hospitals in the Boston area. The CDSC has demonstrated that a CDS engine can be engineered to receive data from, and send reminders to, multiple and non-affiliated health systems using secure protocols in a community cloud [33,34,41-43].

Demonstrations by the CDSC to show that a CDS infrastructure in the cloud can be engineered to securely exchange protected health information is a remarkable achievement that has provided many important lessons [31,33,34,41]. For cloud-based CDS to be widely adopted, it must be shown to be at least as good as traditional approaches to CDS in place locally. Our current study observed considerable agreement between two sets of independently curated sets of reminders. Such agreement suggests that cloud-based CDS infrastructures that enable remote KM and economies of scale are feasible both from an engineering and clinical viewpoint.

Adjustment of Cohen’s Kappa coefficient was necessary due to the potential effects of bias in the CDS logic and prevalence in the relative small sample of patients. Bias can occur when two sets of encoded CDS logic differ in how they assess input data (clinical variables). We hypothesized that independently created and maintained rule logic would potentially assess the patient’s EHR data in different ways. We observed that bias had the greatest effect on Reminder 9, “Due for blood pressure”. Bias increases the Kappa, suggesting that agreement is better than the raw counts indicate. When we adjust for bias, the Kappa coefficient is lower, providing a more realistic impression of the amount of agreement.

The value of Kappa is also affected by the relative probabilities of “Yes” or “No”. We hypothesized that in our limited sample of patients some reminders would be rarely triggered, affecting the probability of a “Yes” versus a “No”. We observed that prevalence had the greatest effect for Reminder 3, “Recent A1c was over 8”. This reminder was rarely triggered, because it required finding a markedly elevated A1c test value older than 3 months but more recent than 5 months. For such low-prevalence events, although the P0 is reasonable (0.95), the initial calculation of Kappa is low (0.28). Adjusting for the low prevalence produces a higher value (PABAK = 0.91) which conveys a more accurate impression of agreement.

Adjusting for prevalence and bias improved agreement for nearly all of the measures. The adjustment revealed that for 7 of the 11 measures there was near-perfect agreement (0.81-1) with 2 measures demonstrating substantial agreement (0.61-0.80), one measure demonstrating moderate agreement (0.41-.060), and one measure demonstrating fair agreement (0.21-0.40). These results are positive, but they also suggest some discordance. Discordance was likely to occur given variation in knowledge engineering techniques as described in prior work [44]. We identified four types of discrepancies between the local and cloud-based services that likely contributed to the discordance: 1) terminology misalignment, 2) local practice variation, 3) temporal windows, and 4) use of exclusions in guidelines implementation. We now examine these discrepancies, which suggest future opportunities for research and development to advance CDS systems.

Terminology misalignment has potential to cause disagreement between two sets of decision support rules, even when operating on the same patient’s data. Of the eleven rules in our project, blood pressure reminders generated the least agreement. The logic of the blood pressure reminder seems very simple: a recommendation to check blood pressure for those adults who do not have a blood pressure documented during the past 12 months. Yet it illustrates a key challenge of computerized implementation of a simple CDS rule. In its initial implementation, the CDSC rules engine only recognized the LOINC code for “Systolic Blood Pressure” (8480–6). Eskenazi Health outpatient clinics measure blood pressure, but the local electronic health record stores blood pressure values using a different LOINC code: “Systolic Blood Pressure – Sitting” (8459–0). These outpatient blood pressure measurements were not recognized by the CDSC engine. Subsequently, the CDSC rules engine was reconfigured to recognize a broader set of codes. This example illustrates that subtle terminology differences (two LOINC codes which almost mean the same thing) can determine whether two engines generate the same advice or not.

Local practice variations also have potential to introduce discrepancies. We reviewed some of the SNOMED CT codes used to represent diagnoses. For example, a young patient without Coronary Artery Disease (CAD) generated a CDSC recommendation to start anti-platelet therapy with aspirin, as if he needed treatment of CAD. Upon review of the patient’s medical history, we found the patient was treated for chest pain due to a gunshot wound. The CCD sent to the CDSC web service included the SNOMED CT code 194828000 (Angina). The CDSC rules engine recognized this SNOMED CT code as an indicator of CAD, and sent a recommendation for anti-platelet therapy. The local CARE rules service did not consider Angina to be a strong indicator of CAD, and thus did not generate any reminder.

The inclusion of more SNOMED CT codes can also have the opposite effect and make a reminder more specific. For example, CARE rules consider anti-platelet medications contraindicated in the setting of Bleeding Disorder, Thrombocytopenia, and GI Bleed. CDSC rules also look for these contraindications, but include additional contraindications too, such as: Esophageal Varices, Coagulation Factor Deficiency Syndrome, and Cerebral Hemorrhage. By searching for these additional SNOMED-CT codes, the CDSC rules might uncover additional contraindications, and thus better suppress inappropriate reminders for anti-platelet therapy.

An under-recognized source of discrepancy arises when different rules query for data from different time ranges. For example, the CDSC rule queries lab data for evidence of microalbuminuria to justify generating a recommendation to start an ACE Inhibitor medication. This rule only looks at a 12 month time frame when searching for this data. On the other hand, the CARE rule does not stop at 12 months. It does not specify any time limit. Older lab data may be included, potentially decreasing the specificity of this reminder.

Important issues arise when checking the existence of Diabetes. The CDSC diabetes classification excludes Gestational Diabetes from the diagnosis of Diabetes, and thus does not send reminders for eye exams or foot exams to women who have only experienced Gestational Diabetes. The CARE rule does not make this exclusion. The CDSC rule asserts Diabetes based only on the patient’s problem list. The CARE rule uses additional criteria to define Diabetes: the use of any oral hypoglycemic medications or insulins from a manually assembled list. The CARE rule also queries hospital ICD9 discharge diagnoses for evidence of diabetes; the CDSC rule does not.

One of the finer points of decision support is the judicious use of exclusions to prevent over-alerting and alert fatigue. For example, the CDSC rule recommends microalbuminuria screening, but excludes patients who already carry a diagnosis of established renal disease. The CARE rule makes no such exclusion; even if a patient has end-stage renal disease, a screening reminder will be generated if no test in such a category has been performed in the last 12 months. The CARE rule only looks for one contraindication to the use of an ACE Inhibitor: an allergy to this class of drugs. The CDSC rule also excludes patients with pregnancy or hyperkalemia. When recommending annual eye exams, only the CARE rule excludes patients with blindness, or patients who have visited the eye clinic during the year; the CDSC rule does not.

Discordance and the discrepancies likely to have contributed to it illustrate an important dichotomy between universal (or cloud-based) CDS versus local CDS knowledge and maintenance. While cloud-based CDS is likely to produce efficiency and cost benefits to health systems, there will likely be a natural loss of control over the implementation and management of CDS which embodies local knowledge and work practices. This may be an anathema to many clinicians who value both the art and science of medicine. However, customization would erode the economies of scale afforded by cloud-based CDS.

Instead of conceptualizing local practice as something that should be accommodated, initiatives like the CDSC should see local variation and terminology development as an opportunity to improve the collective, universal CDS. As new members are integrated, positive deviance should be identified and adapted for the use of the whole community. For example, identifying variant LOINC codes for blood pressure and exclusions such as blindness for diabetic annual eye exam reminders should be welcomed to improve the knowledge base and rule logic for all. If this is the approach taken, then terminologies become aligned and rules become refined over time and the universal CDS becomes more specific and reduces alert fatigue.

Previous studies have shown that guidelines advanced by national and international professional societies are almost never implemented as intended [45]. Often this is due to poorly designed guidelines with vague definitions of the target population or unclear exclusion criteria. Yet sometimes clinical leaders choose to deviate from guidelines due to local habits. While it does not make sense for a cloud-based CDS to customize its rule sets for individual institutions, it may be appropriate for local institutions to adapt the output of the service to meet local needs. The output of the CDSC is a set of reminders that fired for a given input. Local sites have control over how the information is displayed to clinical users, so output from the CDSC could be presented as a non-interruptive alert instead of an interruptive alert, or ignored altogether, depending on local preferences or practices. While designing such customization for every rule might defeat the purpose of cloud-based CDS, it may be appropriate under certain conditions based on local users’ needs, habits or desires.

### Limitations

Our study is chiefly limited by its small size. As the CDSC system was in its initial stage of deployment, just eleven preventive care reminders were implemented. Only the results delivered in the course of 405 patient visits, over a 3 month time period, were analyzed. While we adjusted Kappa to account for prevalence, larger trials comparing local versus cloud-based services would provide greater evidence on the agreement between disparate CDS systems. Further expansion of the CDSC may also uncover other challenges which may lead to more disagreement between the two sets of reminders.

Another limitation is the relative simplicity of the 11 reminders implemented in the study. This set of reminders is not as complex as some rule sets described in the CDS literature. Future plans for the CDSC include implementation of additional preventative rules, including guidelines for immunization schedules and management of chronic illnesses. More complex rule logic, additional exclusion criteria, and rules that rely on social or lifestyle data which are more challenging to extract from electronic health records could pose additional challenges for a remote CDS service. We don’t anticipate that the KM or rule execution of more complex guidelines would be much different than what is presented here, but greater complexity may cause greater discordance with locally developed CDS as more opportunity for diversion from a common standard exists.

Another limitation is the mix of patients in our study sample. As Table 2 indicates, there were small statistically significant differences between the study patients and the larger clinic population, with respect to age and insurance coverage. This is not surprising, because study patients were associated with a convenience set of three physicians, and were not selected at random across multiple sites within the health system. In our judgment, patient demographics are still reasonably characteristic of the larger clinic population. Another, more relevant question is whether our results are generalizable to other outpatient settings in other locations. Our patients are drawn from the urban population of Indianapolis, with a low rate of commercial health insurance. Other institutions elsewhere may serve a very different community. Nevertheless, we believe that our lessons learned about the challenges of data sharing are of great interest regardless of social or economic settings.

## Conclusion

The potential of having one CDS engine providing advice through the cloud to multiple institutions running a variety of EHR systems compels us to further develop and evaluate the CDSC. These results should also encourage research and development by others towards more universal approaches to CDS that can provide economies of scale while delivering relevant knowledge to clinicians at the point-of-care. The development of more integrated web-based services for CDS that build on the international efforts occurring within HL7 would not only strengthen the CDSC but enable other regions and nations to advance CDS knowledge management and services. Efforts to further standardize or align terminologies for common preventative services would support greater harmonization across CDS service efforts nationally and internationally. Finally, improved processes for translating guidelines into executable logic would support cloud-based CDS by enabling better pooling of guideline knowledge and rule sets. These efforts would advance core CDS capabilities as well as cloud-based models to deliver accordant, valuable advice to resource-challenged health care providers across the United States and around the world.

## Abbreviations

CAD: Coronary artery disease; CCD: Continuity of care document; CDS: Clinical decision support; CDSC: Clinical decision support consortium; CDSS: Clinical decision support system; CPOE: Computerized provider order entry; EHR: Electronic health record; KM: Knowledge management; PABAK: Prevalence adjusted bias adjusted Kappa.

## Competing interests

All authors have no competing interest to declare.

## Authors’ contributions

BED and LS contributed to the concept of the paper. SMP supported the statistical analysis. All authors (1) drafted the paper or revised it critically for important intellectual content; and (2) have given their final approval of the submitted paper.

## Pre-publication history

The pre-publication history for this paper can be accessed here:

http://www.biomedcentral.com/1472-6947/14/31/prepub
